# CrossDF: improving cross-domain deepfake detection with deep information decomposition

**DOI:** 10.3389/fdata.2025.1669488

**Published:** 2025-11-18

**Authors:** Shanmin Yang, Hui Guo, Shu Hu, Bin Zhu, Ying Fu, Siwei Lyu, Xi Wu, Xin Wang

**Affiliations:** 1 Computer Science and Technology, Chengdu University of Information Technology, Chengdu, China; 2 University at Buffalo, State University of New York (SUNY), Buffalo, NY, United States; 3 Purdue University, West Lafayette, IN, United States; 4 Microsoft Research Asia, Beijing, China; 5 University at Albany, State University of New York (SUNY), Albany, NY, United States

**Keywords:** deepfake detection, deep information decomposition, model generalization, decorrelation learning, cross-dataset

## Abstract

Deepfake technology represents a serious risk to safety and public confidence. While current detection approaches perform well in identifying manipulations within datasets that utilize identical deepfake methods for both training and validation, they experience notable declines in accuracy when applied to cross-dataset situations, where unfamiliar deepfake techniques are encountered during testing. To tackle this issue, we propose a Deep Information Decomposition (DID) framework to improve Cross-dataset Deepfake Detection (CrossDF). Distinct from most existing deepfake detection approaches, our framework emphasizes high-level semantic attributes instead of focusing on particular visual anomalies. More specifically, it intrinsically decomposes facial representations into deepfake-relevant and unrelated components, leveraging only the deepfake-relevant features for classification between genuine and fabricated images. Furthermore, we introduce an adversarial mutual information minimization strategy that enhances the separability between these two types of information through decorrelation learning. This significantly improves the model's robustness to irrelevant variations and strengthens its generalization capability to previously unseen manipulation techniques. Extensive experiments demonstrate the effectiveness and superiority of our proposed DID framework for cross-dataset deepfake detection. It achieves an AUC of 0.779 in cross-dataset evaluation from FF++ to CDF2 and improves the state-of-the-art AUC significantly from 0.669 to 0.802 on the diffusion-based Text-to-Image dataset.

## 1 Introduction

Recent advances in deep generative models, exemplified by Face2Face (Thies et al., 2016), DeepFake (Rossler et al., 2019), and generative adversarial networks (GANs) (Karras et al., 2019), have significantly elevated the visual realism of synthetic facial imagery. While these technologies hold promise in creative and educational domains, their potential for malicious use poses substantial threats to digital security and undermines public trust. A fundamental challenge in current deepfake detection research lies in the pronounced performance degradation encountered in cross-dataset scenarios, where models trained on one forgery technique fail to generalize to others due to domain shift and overfitting to method-specific artifacts.

In response to this challenge, this paper aims to enhance the generalization capability of deepfake detection across diverse manipulation methods. Our primary contribution is a novel framework that explicitly disentangles deepfake-related features from irrelevant technique-specific information and irrelevant identity-related variations, thereby addressing the critical problem of model overfitting to technique-specific artifacts. This approach represents a significant departure from existing methods and offers a more robust solution for real-world deployment.

Although substantial efforts have been made toward detecting deepfakes in recent years and promising performance have been achieved in intra-dataset settings where both training and testing images are generated using the same deepfake technique. Most of the existing approaches rely on identifying specific visual artifacts produced during the deepfake generation process, such as inconsistencies in blending boundaries between genuine and manipulated faces (Li L. et al., 2020), deviations in head poses (Yang et al., 2019), affine face warping distortions (Li and Lyu, 2019), eye-state anomalies (Li et al., 2018), spectral discrepancies (Li et al., 2021), inter-frame illumination inconsistency (Zhu et al., 2024). Consequently, these methods tend to overfit to the unique artifacts of a particular deepfake technique and exhibit limited generalization capability when exposed to unseen manipulation techniques or datasets. For example, the detector proposed by Qian et al. (2020) achieves an AUC score of 0.98 when trained and tested within the same FaceForensics++ (FF++) deepfake dataset (Rossler et al., 2019), but its performance drops significantly to 0.65 (Nadimpalli and Rattani, 2022; Kim and Kim, 2022) when the model is evaluated on the Celeb-DF dataset (Li Y. et al., 2020) under cross-dataset protocols.

**Figure 1:**
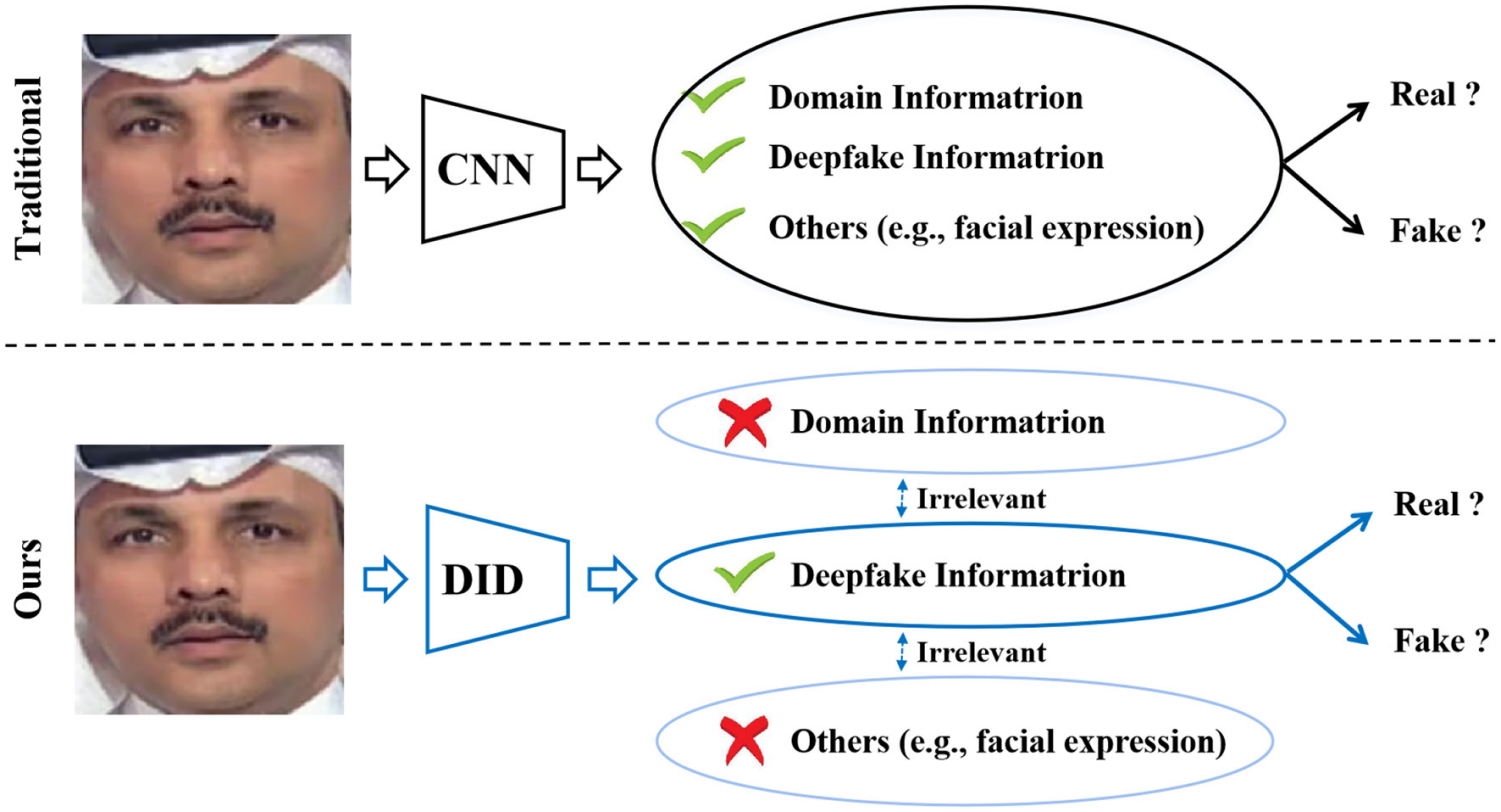
Various information changes entangled with the deepfake information in traditional methods **(top)** would affect real/fake classification accuracy, leading to a sharp degradation in performance when the discrepancies in these components between the training and test sets are more significant than the differences between real and deepfake information. Our deep information decomposition (DID) method **(bottom)** separates the deepfake information from various information irrelevant to real/fake classification to improve the robustness of deepfake detection (FF++ dataset image adapted with permission from Rossler, A., 2019, https://www.kaggle.com/datasets/xdxd003/ff-c23, licensed under CC-BY-NC).

In analyzing the issue of cross-dataset performance degradation, we observe that deepfake detection constitutes a form of fine-grained image classification. As deepfake generation techniques continue to evolve, the discrepancies between authentic and manipulated images have become increasingly subtle, often more nuanced than the variations among deepfakes produced from the same source image using different forgery methods. Furthermore, features directly extracted with conventional deep neural networks (e.g., EfficientNet Tan and Le, 2021) from deepfake images often encapsulate entangled representations that intertwine forgery-related artifacts, domain-specific attributes of the manipulation method, and identity-related factors such as facial expressions and appearance. As illustrated in Figure 1, this feature entanglement exacerbates the sensitivity of detection models to variations in irrelevant factors, particularly those that dominate the representation, thereby impairing generalization across domains.

**Figure 2:**
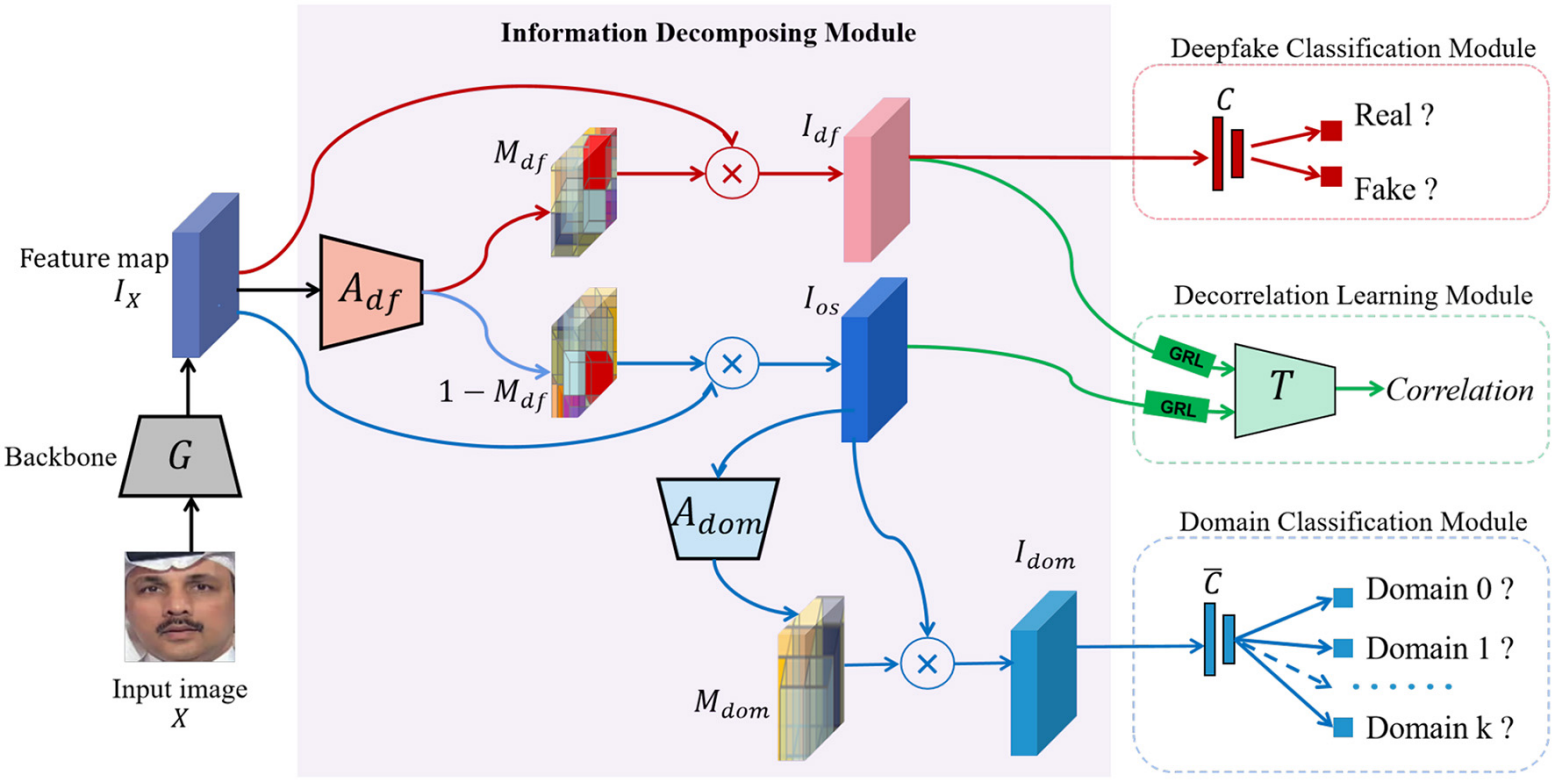
Overview of our Deep Information Decomposition (DID) framework: the feature map IX of an input facial image *X*, generated by a backbone network *G*, is adaptively split into deepfake information *I*_*df*_ and non-deepfake information *I*_*os*_, under the guidance of the deepfake attention network *A*_*df*_ and the supervision of the deepfake classification module (FF++ dataset image adapted with permission from Rossler, A., 2019, https://www.kaggle.com/datasets/xdxd003/ff-c23, licensed under CC-BY-NC). The domain attention network *A*_*dom*_ and the domain classification module extract the forgery method-related (domain) information *I*_*dom*_, ensuring that *I*_*dom*_ is included in the non-deepfake information *I*_*os*_ but being excluded from the deepfake information. Additionally, the decorrelation learning module enforces no overlap between deepfake information and non-deepfake information. This module consists of an information estimation network *T*, which functions in a max-min manner with the information decomposition module through the gradient reversal layer (GRL). *C* and *C* are the deepfake and domain classifiers, respectively.

Inspired by these insights, we propose a Deep Information Decomposition (DID) framework for cross-dataset deepfake detection, as illustrated in Figure 2. Unlike traditional methods that rely on low-level visual artifacts, our approach emphasizes high-level semantic features to capture more generalized forgery cues. Specifically, we regard face images generated by different deepfake techniques, such as Face2Face (Thies et al., 2016) and DeepFake (Rossler et al., 2019), as distinct data domains, thereby formulating cross-dataset detection as a domain generalization problem.

Within the proposed framework, facial representations are adaptively decomposed into three semantically distinct components: deepfake-related information, which captures universal manipulation traces common across different forgery methods; irrelevant technique-specific information, which encodes distinctive artifacts attributable to particular generation approaches such as Face2Face (Thies et al., 2016) and DeepFake (Rossler et al., 2019); and irrelevant identity-related variations, including facial expressions, lighting conditions, and personal identity attributes. This decomposition is achieved through two dedicated attention modules that learn to selectively emphasize and isolate these components in a self-supervised manner. Crucially, only the deepfake-relevant features are utilized during the authentic-versus-fake classification phase, effectively suppressing the influence of extraneous factors and technique-specific biases. To further enhance feature discriminability and domain invariance, we introduce a decorrelation learning module that minimizes mutual information between the deepfake-specific features and both types of irrelevant variations (identity-related and technique-specific) via adversarial training. This explicitly encourages statistical independence among feature components, thereby significantly improving the model's robustness and generalization capability across unseen datasets and diverse manipulation techniques. Extensive experiments on multiple benchmarks demonstrate the effectiveness and superiority of our framework in cross-dataset deepfake detection scenarios.

In summary, our main contributions are:

1. We propose a novel end-to-end Deep Information Decomposition (DID) framework. It formulates cross-dataset deepfake detection as a domain generalization problem and decomposes face image information into deepfake-related, technique-specific, and identity-related information components to enhance generalization.

2. We introduce a decorrelation learning module that promotes the independence of the deepfake-related information from all irrelevant variations (identity-related and technique-specific) without requiring knowledge of their distribution functions or relationships, thereby enhancing the robustness of deepfake detection.

3. We conducted extensive experiments that demonstrated the superiority of our framework, achieving state-of-the-art performance on the challenging cross-dataset deepfake detection task.

## 2 Related work

This section provides a brief review of deepfakes, cross-dataset deepfake detection, and information decomposing. For more details about the deepfake techniques and deepfake datasets, please refer to Nguyen et al. (2022).

### 2.1 Deepfakes

Deepfake, broadly referring to manipulated or synthetic media that convincingly mimics natural content (Nguyen et al., 2022), poses a significant threat to digital media integrity. This paper focuses specifically on deepfake faces. Existing creation methods can be broadly classified into two categories: transfer-based and synthesis-based approaches.

Transfer-based deepfake methods manipulate target faces by transferring facial attributes (e.g., expression, mouth movement) or entire identities from a reference source. The primary benefit of these methods is their ability to produce highly convincing forgeries by leveraging real human features, making the manipulations contextually consistent and realistic. For instance, Face2Face (Thies et al., 2016) enables real-time facial reenactment by transferring expressions from a source to a target video, preserving the target's identity. FaceSwap and DeepFake (Rossler et al., 2019) replace the face region in a target video with a source face, often relying on autoencoders to learn and swap identity attributes. Neural Textures (Thies et al., 2019) combines learned neural textures with deferred rendering to improve the realism of synthesized facial motions, particularly around critical areas like the mouth. Similarly, Bao et al. (2018) decomposes faces into identity and attribute representations, allowing controlled attribute transfer while retaining original identity. However, a key limitation of these methods is their reliance on blending operations and warping, which often introduce subtle artifacts. These can manifest as inconsistencies in lighting, blurring at blending boundaries, misaligned facial geometry, or unnatural eye movements. While these artifacts form the basis for many early detection algorithms, they also represent a point of vulnerability for forgers: as generation models improve, these artifacts become increasingly subtle, making detection more challenging.

Synthesis-based deepfake methods, in contrast, generate entirely novel facial images or attributes without direct reference to a specific source individual. This category is dominated by Generative Adversarial Networks (GANs) and variants of 3D Morphable Models (3DMM). The main advantage of these approaches is their ability to create highly diverse and novel forgeries that do not rely on the availability of a reference face/video, expanding the scope of potential attacks. For example, 3DMM-guided approaches (Geng et al., 2019), which generate arbitrary facial expressions and viewpoints under structured geometric control, enhancing pose robustness. StyleGAN (Karras et al., 2019), which produces high-fidelity, diverse facial imagery through its style-based generator, significantly raising the visual quality bar for synthetic faces. GANprintR (Neves et al., 2020), which is specifically designed to generate realistic deepfakes while attempting to evade detection by minimizing known GAN fingerprints. Despite their high visual quality, synthesis-based methods can introduce their own unique artifacts. These include frequency domain abnormalities (e.g., spectral disparities), physiological implausibilities (e.g., asymmetric pupils), and inherent fingerprints left by the generator architecture itself. Nevertheless, the rapid advancement of these technologies has led to a continuous reduction of such artifacts, rendering detection strategies that rely on them increasingly obsolete.

### 2.2 Cross-dataset deepfake detection

The paramount challenge in contemporary deepfake detection is generalization, the ability of a model to perform robustly on forgeries generated by unseen methods or datasets. While numerous detection methods (Zhao H. et al., 2021; Shiohara and Yamasaki, 2022; Dong et al., 2022) achieve impressive performance in intra-dataset scenarios, they suffer from catastrophic performance degradation under cross-dataset evaluation. This failure mode primarily stems from models overfitting to technique-specific artifacts (e.g., blending patterns of FaceSwap, frequency signatures of a specific GAN) rather than learning a universal representation of “forgery”. In response to this generalization challenge, research has evolved along several key directions: Data Augmentation for Domain Expansion, a straightforward strategy is to augment training data to simulate a wider variety of forgery types, thereby encouraging the model to learn more invariant features. For instance, Zhao T. et al. (2021) proposed dynamic data augmentation strategies to artificially expand the diversity of training samples, effectively exposing the model to a broader spectrum of potential artifacts. Nadimpalli and Rattani (2022) advanced this concept by employing a reinforcement learning-based strategy to intelligently select augmentation policies, mitigating domain shift more effectively than random strategies. A core issue with these approaches is that they rely on synthetic augmentations which may not fully capture the complex and realistic distribution of novel deepfake techniques, potentially limiting their effectiveness against truly advanced unseen forgeries.

Inherent Forensic Feature Learning, another line of work seeks to identify and leverage common, intrinsic traces left by deepfake generation processes that are theoretically invariant across different methods. Kim and Kim (2022) focused on color distribution inconsistencies introduced during the face-synthesis process, a low-level cue that is often shared across different manipulation techniques. Yu et al. (2022) aimed to learn and amplify common forgery features that persist across diverse datasets, moving beyond dataset-specific biases. The fundamental challenge here is that as generative models become more advanced, they produce fewer inherent artifacts. This makes the discovery of robust, shared forensic features increasingly difficult.

Explicit Domain Alignment and Bridging, the most directly relevant approaches explicitly model and aim to reduce the distributional gap between different deepfake domains. Yu et al. (2023) made significant strides by using Adaptive Normalization layers and generating “bridging samples” to create a continuous latent space between domains, explicitly narrowing the distribution gap for improved generalization. Similarly, Yin et al. (2024) employed a framework based on Invariant Risk Minimization (IRM), designed to prioritize domain-invariant features and aligned representations, thus enhancing cross-domain performance. Huang et al. (2023) introduced a video-level contrastive learning framework to maintain feature consistency across varying compression levels, a critical step toward real-world applicability where compression is ubiquitous. While effective, many of these methods can introduce significant algorithmic complexity (e.g., requiring multiple domains during training, generative modules for sample synthesis, or complex loss functions), which may hinder their practical deployment and scalability.

While existing research has made valuable progress through data augmentation, feature learning, and domain alignment, the problem remains largely open. Many methods still struggle with the sheer diversity and evolving nature of deepfake techniques, often requiring complex multi-domain training or failing to generalize to the next generation of generators. This underscores the need for a more elegant and principled approach to learning domain-agnostic forgery features. Our proposed method addresses this by explicitly disentangling deepfake-related features from irrelevant variations, aiming to isolate a more pure and generalizable representation of manipulation that is invariant to the creation method.

### 2.3 Information decomposing

Information decomposition, which aims to disentangle complex and intertwined data into distinct, semantically meaningful components and isolate those relevant to specific tasks, has been widely adopted across various computer vision applications. For instance, methods such as those proposed by Tran et al. (2017) and Wang et al. (2019) separate identity-related features from pose and age variations, respectively, thereby reducing the influence of these factors in face recognition systems. Similarly, Wu et al. (2019) decomposes facial representations into identity and modality components to improve performance in NIR-VIS heterogeneous face recognition.

In the domain of deepfake detection, several studies have leveraged disentanglement strategies to enhance generalization and detection accuracy. Hu et al. (2021) detect forgery regions by disentangling multi-scale features and training the detector specifically on these localized artifacts. Liang et al. (2022) separate artifact-related features from content information to minimize the confounding effect of identity and background during detection. More recently, Yu et al. (2024) introduced a framework that progressively disentangles forgery-relevant features from source-related features through multi-view learning, operating from image space to feature space. Likewise, Yan et al. (2023) employed a multi-task learning setup with a conditional decoder to isolate generalizable forgery attributes from those specific to particular generation methods.

In this paper, we propose an information decomposition framework that achieves disentanglement using a complementary attention mechanism, differing from methods such as Hu et al. (2021); Liang et al. (2022); Yan et al. (2023), which achieve information disentanglement through feature encoding and decoding with carefully designed reconstruction losses (e.g., self-reconstruction, cross-reconstruction, and feature reconstruction). Furthermore, we introduce a deep decorrelation module to ensure that the forgery-relevant features used for deepfake detection remain inherently independent of other features. This intrinsic independence is a crucial factor that is frequently neglected in existing literature like Yu et al. (2024), where such independence is not emphasized. Our strategy for achieving feature independence diverges from that of Yan et al. (2023), who employ a contrastive loss to optimize the Euclidean distance between the decoupled features. Our approach enhances the model's robustness and generalization across different forgery techniques and datasets.

## 3 Our method

The pipeline of our proposed method is illustrated in Figure 2. Specifically, for an input image *X*, we employ a CNN-based feature extractor *G* parameterized by θ to capture its representative features, denoted as *I*_*X*_: = *G*(θ; *X*). These extracted features are then decomposed into three components: (1) the deepfake-related representation, *I*_*df*_, which contains the critical information used to detect deepfakes; (2) the domain-related representation, *I*_*dom*_, which captures the characteristics of the forgery technique or method responsible for generating the deepfake; and (3) the remaining representation. The information decorrelation module ensures that the deepfake information *I*_*df*_ is optimized to be independent of other representations, thereby enhancing the performance of the decomposition. The robust deepfake classification module is designed to train a model capable of classifying deepfakes effectively, even in imbalanced datasets, thus enhancing the model's generalization ability. Additionally, the domain classification module is intended to identify the domain to which *I*_*dom*_ belongs. Before diving into the details of these modules, we introduce some commonly used notations.

### 3.1 Notation

Our method takes images from existing deepfake datasets as input data. Let $S={\left\{\left(X_{i},Y_{i},D_{i}\right)\right\}}_{i=1}^{n}$ be a training dataset that contains images $X_{i}\in {\mathbb{R}}^{d}$ and their corresponding labels *Y*_*i*_∈{0, 1}, where 0 denotes real and 1 indicates fake. $D_{i}:={\left[D_{i}^{0},D_{i}^{1},...,D_{i}^{k}\right]}^{⊤}$ represents the domain label of *X*_*i*_, where the domain size of fake data *k*≥1 and $D_{i}^{j}\in \left\{0,1\right\},\forall j\in \left\{0,1,...,k\right\}$. In particular, $D_{i}^{j}=1$ indicates that *X*_*i*_ is from the *j*-th domain. Specifically, *X*_*i*_ is from the real data domain if *j* = 0 and from the fake data domain *j* (i.e., forged by the method *j*) if *j*>0. For example, the fake images in the FF++ dataset (Rossler et al., 2019) are generated by four face manipulation methods: Deepfakes, Face2Face, FaceSwap, and NeuralTextures. Therefore, *k* = 4. In this work, we assume each image comes from a single domain.

### 3.2 Information decomposition module

Motivated by Yang et al. (2021), the information decomposition module consists of a deepfake attention network *A*_*df*_ parameterized by ψ [denoted as *A*_*df*_(ψ; .)] and a domain attention network *A*_*dom*_ parameterized by φ [denoted as *A*_*dom*_(φ; .)], as shown in Figure 2. Taking the face information *I*_*X*_ embedded with entangled information as input, the deepfake attention network focuses on deepfake-relevant information, thereby it decomposes *I*_*X*_ into two complementary components: the deepfake-relevant information *I*_*df*_ and the deepfake-irrelevant information *I*_*os*_. This process can be formulated as follows,


$$\begin{array}{l} M_{df}=A_{df}\left(\psi ;I_{X}\right),\\ I_{df}=M_{df}⊗I_{X},\\ I_{os}=\left(1-M_{df}\right)⊗I_{X}, \end{array}$$ (1)

where $M_{df}\in {\left[0,1\right]}^{c\times h\times w}$ is the deepfake-relevant information attention map; ⊗ represents the Hadamard product.

After receiving the deepfake-irrelevant information *I*_*os*_, the domain attention network *A*_*dom*_ focuses on extracting and modeling explicitly forgery technique information. It decomposes the deepfake-irrelevant information *I*_*os*_ into the forgery technique-related information *I*_*dom*_ and others as follows,


$$\begin{array}{l} M_{dom}=A_{dom}\left(\varphi ;I_{os}\right),\\ I_{dom}=M_{dom}⊗I_{os}, \end{array}$$ (2)

where $M_{dom}\in {\left[0,1\right]}^{c\times h\times w}$ is the forgery technique-related information attention map.

**Figure 3:**
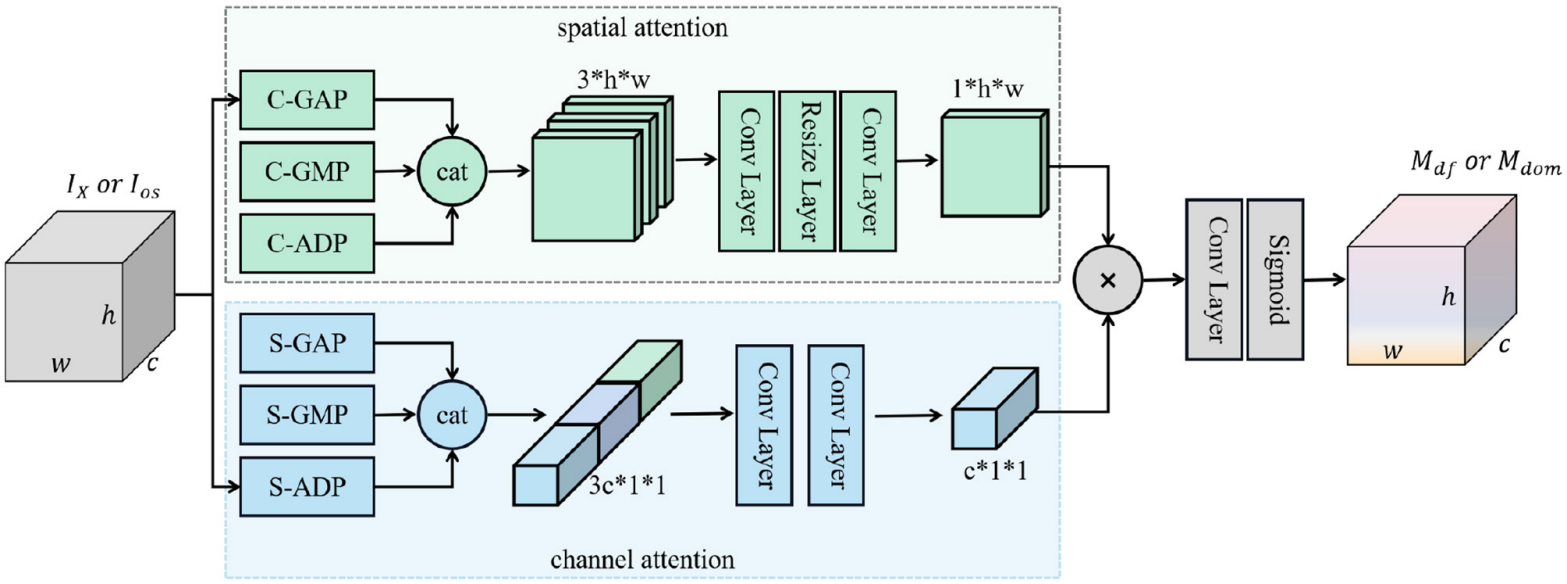
Architecture of the deepfake (domain) attention network. This network takes the face information *I*_*X*_ (deepfake-irrelevant information *I*_*os*_) as input and then learns to produce an attention map that highlights the significance (potential) of the input data being correlated with deepfake-relevant (domain-relevant) information. “cat” means concatenating all input data along the channel dimension; ⊗ represents the Hadamard product; “C-GAP,” “C-GMP,” and “C-ADP” represent cross-channel global average pooling, cross-channel global max pooling, and cross-channel adaptive pooling, respectively; “S-GAP,” “S-GMP,” and “S-ADP” are cross-spatial global average pooling, cross-spatial global max pooling, and cross-spatial adaptive pooling, respectively.

The deepfake attention network *A*_*df*_ and the domain attention network *A*_*dom*_ are constructed to learn attention maps across spatial and channel dimensions concurrently. They share an identical architecture as shown in Figure 3, where each convolution (Conv) Layer is followed by a PReLU (Parametric Rectified Linear Unit, PReLU) activation function. S-ADP is implemented by a channel-wise spatial convolution layer followed by a sum pooling layer, while C-ADP is implemented with a 1 × 1 convolution layer. Both *A*_*df*_ and *A*_*dom*_ are trained to efficiently capture the essential deepfake-related and domain-related information within the input data, respectively. The pseudocode of information decomposition is shown in Algorithm 1.

Algorithm 1 Information decomposition process.

**Input**: Entangled face information *I*_*X*_, deepfake attention network *A*_*df*_(ψ;.), and domain attention network *A*_*dom*_(φ;.)
**Output**: Decomposed components *I*_*df*_, *I*_*dom*_, and *I*_*res*_
1 **Step 1**: Decompose deepfake-relevant information
2 *M*_*df*_←*A*_*df*_(ψ; *I*_*X*_)
3 *I*_*df*_←*M*_*df*_⊗*I*_*X*_
4 *I*_*os*_←(1−*M*_*df*_)⊗*I*_*X*_
5 **Step 2**: Decompose technique-specific information
6 *M*_*dom*_←*A*_*dom*_(φ; *I*_*os*_)
7 *I*_*dom*_←*M*_*dom*_⊗*I*_*os*_
8 *I*_*res*_←(1−*M*_*dom*_)⊗*I*_*os*_

### 3.3 Decorrelation learning module

The disentangled elements (deepfake and non-deepfake information) are anticipated to be partitioned into two separate representations. To accomplish this, orthogonal constraints are commonly applied to these disentangled components (Wang et al., 2019). However, linear dependence/independence can hardly characterize the intricate relationships between deepfake and non-deepfake information in a high-dimensional and non-linear space. In contrast, mutual information (Kinney and Atwal, 2014) (MI) is capable of capturing arbitrary dependencies between any two variables.

With this motivation, we apply mutual information to evaluate dependencies between deepfake information *I*_*df*_ and non-deepfake information *I*_*os*_, formulated as follows:


$$\begin{array}{l} \text{MI}\left(I_{df};I_{os}\right)=D_{\text{KL}}\left(P\left(I_{df},I_{os}\right)||P\left(I_{df}\right)⊗P\left(I_{os}\right)\right), \end{array}$$ (3)

where *P*(·, ·) is the joint probability distribution, *P*(·) denotes the marginal probability distribution, and *D*_KL_ is the Kullback-Leibler divergence (Joyce, 2011).

Since the probability densities *P*(*I*_*df*_, *I*_*os*_) and *P*(*I*_*df*_)⊗*P*(*I*_*os*_) are not known, it becomes challenging to directly minimize MI(*I*_*df*_; *I*_*os*_). Belghazi et al. (2018) introduced a Mutual Information Neural Estimation (MINE) to derive a lower bound on MI's Donsker-Varadhan representation. Subsequently, Hjelm et al. (2019) proposed a Jensen-Shannon MI estimator, which is based on the Jensen-Shannon divergence (Menéndez et al., 1997). This method has been demonstrated to be more stable and yields improved results.

Inspired by Hjelm et al. (2019), we construct a mutual information estimation network *T* with parameterizes ϕ to approximate MI(*I*_*df*_; *I*_*os*_) as follows,


$$\begin{array}{l} \text{MI}(I_{df};I_{os})\geq {\hat{I}}^{JSD}(I_{df};I_{os})\\ \;=E_{x\sim P\left(I_{df},I_{os}\right)}\left[\log\sigma (T(\phi ;x))\right]\\ \;+E_{x\sim P\left(I_{df}\right)⊗P\left(I_{os}\right)}\left[\log\left(1\text{​}-\text{​}\sigma (T(\phi ;x))\right)\right], \end{array}$$ (4)

where σ is the sigmoid function; $T\left(\phi ;\cdot \right):{\mathbb{R}}^{d_{x}}\to \mathbb{R}$ acts as the discriminator function in GANs (*d*_*x*_ is the dimension of *I*_*df*_ and *I*_*os*_), it aims to estimate and maximize the lower bound of MI(*I*_*df*_; *I*_*os*_), while the target of the previously designed information decomposition module (acting as the generator function in GANs) is to minimize the MI value between *I*_*df*_ and *I*_*os*_ to achieve a sufficient separation. Specifically, we have the following learning objectives:


$$\begin{array}{l} {ℒ}_{dec}=\underset{\theta ,\psi }{\min}\;\underset{\phi }{\max}(E_{x\sim P\left(I_{df},I_{os}\right)}\left[\log\sigma (T(\phi ;x))\right]\\ \;+E_{x\sim P\left(I_{df}\right)⊗P\left(I_{os}\right)}\left[\log\left(1\text{​}-\text{​}\sigma (T(\phi ;x))\right)\right]). \end{array}$$ (5)

To implement the aforementioned min-max game using standard back-propagation (BP) training, we incorporate a Gradient Reversal Layer (GRL) (Ganin and Lempitsky, 2015) before the network *T* (as illustrated in Figure 2). During the back-propagation procedure, the GRL modifies the gradient by multiplying a negative scalar, −β, as it passes from the subsequent layer to the preceding layer, where we typically set β∈(0, 1). This technique has also been utilized in various existing works, such as Belghazi et al. (2018); Hjelm et al. (2019). The network *T* consists of three convolution layers, each followed by a ReLU activation function, and concludes with a fully connected (FC) layer.

### 3.4 Deepfake classification module

After extracting deepfake-related information *I*_*df*_, we need to consider how to leverage it for training a deepfake detection model. In existing literature, Binary Cross-entropy (BCE) loss is commonly employed for this purpose. However, it is well-known that the BCE loss lacks robustness against imbalanced datasets. Training models with BCE loss on one specific deepfake dataset can lead to a considerable performance decline when evaluated on a different deepfake dataset (Pu et al., 2022).

Building on this observation, we propose a robust deepfake detection loss aimed at enhancing the generalization ability of the trained model by utilizing deepfake-related information *I*_*df*_ instead of the complete information *I*_*X*_. The AUC metric inspires our loss since it is a robust measure to evaluate the classification capability of a model, especially when facing imbalanced data. Specifically, it estimates the size of the area under the receiver operating characteristic (ROC) curve (AUC; He and Garcia, 2009), which is composed of False Positive Rates (FPRs) and True Positive Rates (TPRs). However, the AUC metric cannot be directly used as a loss function since it is challenging to compute during each training iteration. Inspired by Pu et al. (2022), we use the normalized WMW statistic (Yan et al., 2003), equivalent to AUC, to design our loss function.

Specifically, we define a set of indices of fake instances and real instances as $F=\left\{i|Y_{i}=1\right\}$ and $R=\left\{i|Y_{i}=0\right\}$, respectively. We add a multilayer perceptron (MLP) $C:{\mathbb{R}}^{d_{x}}\to \mathbb{R}$ (*d*_*x*_ is the dimension of *I*_*df*_) parameterized by ω to distinguish fake and real instances, where the input is *I*_*df*_ and the output is a real value. Network *C* predicts input *I*_*df*_ to be fake with probability σ(*C*(ω; *I*_*df*_)). Without loss of generality, *C*(ω; *I*_*df*_) induces the prediction rule such that the predicted label of *I*_*df*_ can be *I*[σ(*C*(ω; *I*_*df*_))≥0.5], where *I*[·] is an indicator function with *I*[*a*] = 1 if a is true and 0 otherwise. For simplicity, we assume $C\left(\omega ;I_{df}^{X_{i}}\right)\neq C\left(\omega ;I_{df}^{X_{j}}\right)$ for any *X*_*i*_≠*X*_*j*_ (ties can be broken in any consistent way), where $I_{df}^{X_{i}}$ represents the deepfake information of the sample *X*_*i*_. Then the normalized WMW can be formulated as follows,


$$\begin{array}{l} \text{WMW}=\frac{1}{\left|F||R\right|}\underset{i\in F}{\sum }\underset{j\in R}{\sum }I\left[C\left(\omega ;I_{df}^{X_{i}}\right)>C\left(\omega ;I_{df}^{X_{j}}\right)\right], \end{array}$$ (6)

where |F| and |R| are the cardinality of F and R, respectively. However, WMW is non-differentiable due to the indicator function, which is the primary obstacle to using it as a loss function. Therefore, we use its alternative version (Yan et al., 2003):


$$\begin{array}{l} L_{\text{AUC}}=\frac{1}{\left|F||R\right|}\underset{i\in F}{\sum }\underset{j\in R}{\sum }E\left(C\left(\omega ;I_{df}^{X_{i}}\right),C\left(\omega ;I_{df}^{X_{j}}\right)\right), \end{array}$$ (7)

with


$$\begin{array}{l} E(C(\omega ;I_{df}^{X_{i}}),C(\omega ;I_{df}^{X_{j}}))\\ :=\text{​​}\{\begin{array}{cc} {\left(-(C(\omega ;I_{df}^{X_{i}})\text{​}-\text{​}C(\omega ;I_{df}^{X_{j}})\text{​}-\text{​}\gamma )\right)}^{p}, & C(\omega ;I_{df}^{X_{i}})-C(\omega ;I_{df}^{X_{j}})<\gamma ,\\ 0, & \text{otherwise}, \end{array} \end{array}$$ (8)

where 0 < γ ≤ 1 and *p*>1 are two hyperparameters. We combine this AUC loss and the conventional BCE loss to construct a learning objective for robust deepfake classification:


$$\begin{array}{l} \;{ℒ}_{cls}=\alpha {ℒ}_{\text{BCE}}+(1-\alpha ){ℒ}_{\text{AUC}}\\ {ℒ}_{\text{BCE}}=-\frac{1}{n}\sum_{i=1}^{n}\left[Y_{i}\cdot \log(\sigma (C(\omega ;I_{df}^{X_{i}}))\right)\\ \;+(1-Y_{i})\cdot \log(1-\sigma (C(\omega ;I_{df}^{X_{i}})))] \end{array}$$ (9)

where α is a hyperparameter designed to balance the weights of the BCE loss and the AUC loss.

### 3.5 Domain classification module

A domain classification module is also designed using another MLP $\bar{C}:{\mathbb{R}}^{d_{I_{X}}}\to {\mathbb{R}}^{k+1}$ parameterized by $\bar{\omega }$ to map the forgery method related-domain information *I*_*dom*_ into a (k+1)-dimensional domain vector. Specifically, we have $\bar{C}\left(\bar{\omega };I_{dom}\right)={\left[{\bar{C}}^{0}\left(\bar{\omega };I_{dom}\right),{\bar{C}}^{1}\left(\bar{\omega };I_{dom}\right),...,{\bar{C}}^{k}\left(\bar{\omega };I_{dom}\right)\right]}^{⊤}$ , where ${\bar{C}}^{j}\left(\bar{\omega };I_{dom}\right)$ is the *j*-th domain prediction. We then apply the softmax function to compute the probability of each domain that *I*_*dom*_ belongs to and combine its domain label to construct a domain classification loss based on the cross-entropy (CE) loss. Therefore, we have


$$\begin{array}{l} L_{dom}=-\frac{1}{n}\overset{n}{\underset{i=1}{\sum }}\overset{k}{\underset{j=0}{\sum }}D_{i}^{j}\log\left(S\left[{\bar{C}}^{j}\left(\bar{\omega };I_{dom}^{X_{i}}\right)\right]\right) \end{array}$$ (10)

where $S\left[{\bar{C}}^{j}\left(\bar{\omega };I_{dom}^{X_{i}}\right)\right]\in \left(0,1\right)$ is the *j*-th domain predicted probability for the domain information of *I*_*X*_*i*__ after using softmax operator *S*[·].

#### 3.5.1 Overall loss

To sum up, the proposed framework is optimized with the following final loss function:


$$\begin{array}{l} L=\lambda_{dec}L_{dec}+\lambda_{cls}L_{cls}+\lambda_{dom}L_{dom} \end{array}$$ (11)

where λ_*dec*_, λ_*cls*_, and λ_*dom*_ are hyperparameters that can balance these loss terms. In practice, the optimization problem in Equation 11 can be solved with an iterative stochastic gradient descent and ascent approach (Beznosikov et al., 2022). Specifically, we first initialize the model parameters θ, ψ, φ, ϕ, ω, and $\bar{\omega }$. Then we alternate uniformly at random a mini-batch $S_{b}$ of training samples from the training set S and do the following steps on $S_{b}$ for each iteration:


$$\begin{array}{l} \left(\begin{array}{c} \theta_{l+1}\\ \psi_{l+1}\\ \varphi_{l+1}\\ \phi_{l+1}\\ \omega_{l+1}\\ {\bar{\omega }}_{l+1} \end{array}\right)=\left(\begin{array}{c} \theta_{l}\\ \psi_{l}\\ \varphi_{l}\\ \phi_{l}\\ \omega_{l}\\ {\bar{\omega }}_{l} \end{array}\right)-\left(\begin{array}{c} \eta_{\theta }\partial_{\theta }L{|}_{\theta =\theta_{l}}\\ \eta_{\psi }\partial_{\psi }L{|}_{\psi =\psi_{l}}\\ \eta_{\varphi }\partial_{\varphi }L{|}_{\varphi =\varphi_{l}}\\ -\eta_{\phi }\beta \partial_{\phi }L{|}_{\phi =\phi_{l}}\\ \eta_{\omega }\partial_{\omega }L{|}_{\omega =\omega_{l}}\\ \eta_{\bar{\omega }}\partial_{\bar{\omega }}L{|}_{\bar{\omega }={\bar{\omega }}_{l}} \end{array}\right), \end{array}$$ (12)

where L is defined on $S_{b}$, η_θ_, η_ψ_, η_φ_, η_ϕ_, η_ω_, and $\eta_{\bar{\omega }}$ are learning rates, and $\partial_{\theta }L$, $\partial_{\psi }L$, $\partial_{\varphi }L$, $\partial_{\phi }L$, $\partial_{\omega }L$, and $\partial_{\bar{\omega }}L$ are the (sub)gradient of L with respect to θ, ψ, φ, ϕ, ω, and $\bar{\omega }$. In the testing phase, we only use the feature extractor *G*, attention module *A*_*df*_, and the deepfake classification module *C*. The pseudocode is shown in Algorithm 2.

Algorithm 2 Deep information decomposition. 
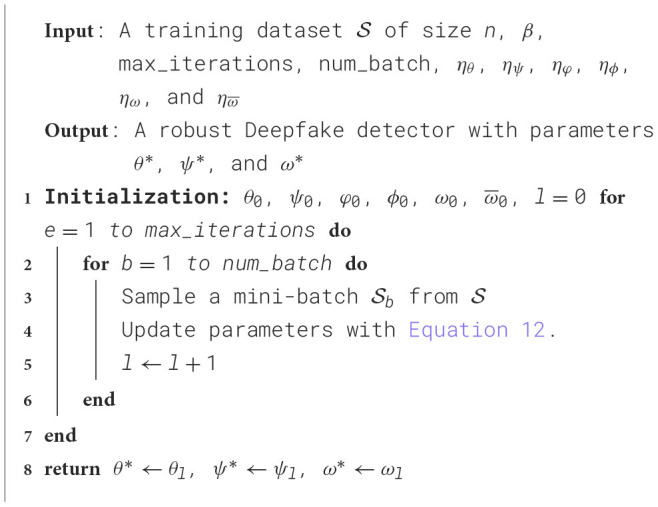

## 4 Experiments

This section evaluates the effectiveness of the proposed DID framework in terms of cross-dataset deepfake detection performance. In the following discussion, we will exchange the “method” or “framework” used for DID.

### 4.1 Experimental settings

#### 4.1.1 Datasets

For fair comparisons with state-of-the-art methods, we adopt the two most widely used datasets, FF++ (Rossler et al., 2019) and Celeb-DF (Li Y. et al., 2020), in our experiments. Specifically, the high-quality (HQ, compressed with a constant rate factor of 23) version of FF++ is utilized throughout all experiments. It includes one real video subset and four fake video subsets generated using FaceSwap, DeepFakes, Face2Face, and Neural Textures techniques, respectively. Each subset contains 1,000 videos, split into 720 for training, 140 for validation, and 140 for testing (Rossler et al., 2019). The Celeb-DF (Li Y. et al., 2020) dataset contains real and fake videos of 59 celebrities. Following the official protocols in Li Y. et al. (2020), we use the latest version, Celeb-DF V2 (CDF2), which includes 590 real celebrity (Celeb-real) videos, 300 real videos from YouTube (YouTube-real) and 5,639 synthesized celebrity (Celeb-synthesis) videos generated from Celeb-real.

#### 4.1.2 Compared methods and evaluation metrics

To assess the performance of our framework, we benchmark it against the following state-of-the-art (SOTA) frame-level baseline methods: F^3^-Net (Qian et al., 2020), CFFs (Yu et al., 2022), RL (Nadimpalli and Rattani, 2022), Multi-task (Nguyen et al., 2019), Two Branch (Masi et al., 2020), MDD (Zhao H. et al., 2021), and NoiseDF (Wang and Chow, 2023). The results of Multi-task and Two Branch are drawn from Nadimpalli and Rattani (2022), while these for MDD are referenced from Yu et al. (2022). We evaluate the methods using two commonly used metrics: the area under the receiver operating characteristic curve (AUC) and the equal error rate (EER), both of which are standard in previous works for performance comparison.

#### 4.1.3 Implementation details

In our experiments, we utilize EfficientNet v2-L (Tan and Le, 2021) pre-trained on the ImageNet dataset as the backbone for feature extraction. All face images are aligned to a size of 224 × 224 using the MTCNN method (Zhang et al., 2016), and subsequently converted from RGB to grayscale before being fed into the proposed framework. The model is trained using the Adam optimizer with a weight decay of 5*e*^−4^ and a learning rate of 1*e*^−5^. We set the learning rate η_ψ_, η_φ_, η_ϕ_, η_ω_, and $\eta_{\bar{\omega }}$ in Equation 12 to be 10 times that of η_θ_ ($\eta_{\theta }=1e^{-5}$). The batch size is set to 15, and each epoch consists of 6000 iterations. We set γ and *p* in Equation 8 to 0.15 and 2, respectively. We use α = 0.5 in Equation 9. The hyperparameters λ_*cls*_, λ_*dom*_, and λ_*dec*_ in Equation 11 are set to 1, 1, and 0.01, respectively. The hyperparameter β in Equation 12 is adapted to increase from 0 to 1 in the training procedure as β = 2.0/(1.0+*e*^−5*p*^)−1.0, where *p* is the ratio of the current training epochs to the maximum number of training epochs. All experiments are conducted on two NVIDIA RTX 3080 GPUs, using Pytorch 1.10 and Python 3.6. In all our experiments, no data augmentation techniques, such as random image compression, image flip, and brightness contrast, are employed.

### 4.2 Intra-dataset evaluation

We assess the detection performance of our proposed method, DID, in the intra-dataset scenario, where both the training and testing datasets are derived from the FF++ dataset and are disjoint from one another. Table 1 presents the results of the intra-dataset evaluation along with comparisons to the baseline methods. We can see that our method achieves 0.970 on AUC, surpassing the performance of Multi-task (Nguyen et al., 2019), Two Branch (Masi et al., 2020), and NoiseDF (Wang and Chow, 2023) methods. Additionally, it demonstrates competitiveness with the leading performance, which is an AUC score of 0.998 attained by the MDD method (Zhao H. et al., 2021).

**Table 1:** Intra-dataset evaluation on FF++ and cross-dataset evaluation from FF++ to CDF2.

**Methods**	**Intra-dataset**	**Cross-dataset**
	**AUC** ↑	**AUC** ↑
Xception (Rossler et al., 2019)	0.997	0.653
Multi-task (Nguyen et al., 2019)	0.763	0.543
Two Branch (Masi et al., 2020)	0.931	0.734
MDD (Zhao H. et al., 2021)	**0.998**	0.674
RL (Nadimpalli and Rattani, 2022)	0.994	0.669
F^3^-Net (Qian et al., 2020)	0.981	0.651
CFFs (Yu et al., 2022)	0.976	0.742
NoiseDF (Wang and Chow, 2023)	0.940	0.759
FDML (Yu et al., 2024)	0.996	0.731
DIRE (Wang et al., 2023)	0.994	-
DID (Ours)	0.970	**0.779**

The highest results are highlight in bold.

### 4.3 Cross-dataset evaluation

#### 4.3.1 Cross-dataset evaluation

The cross-dataset generalization performance of the proposed method and comparison with the baselines are also shown in Table 1. All models are trained on the FF++ dataset and subsequently evaluated on the unseen CDF2 dataset. Results indicate that all methods suffer significant performance degradation in this challenging cross-dataset scenario when compared to the intra-dataset scenario. For instance, MDD (Zhao H. et al., 2021) declines from 0.998 to 0.674 AUC. In contrast, our DID method demonstrates impressive generalization capability, outperforming the CFFs (Yu et al., 2022) and NoiseDF (Wang and Chow, 2023) methods by margins of 4.99% (0.779 vs. 0.742) and 2.635% (0.779 vs. 0.759) respectively in terms of AUC. These results confirm the effectiveness and superiority of our framework.

#### 4.3.2 Generalization on diffusion-generated facial forgery dataset

To further assess the generalization performance of our proposed method, we conduct deepfake detection on the diffusion-generated facial forgery dataset, which presents a significant challenge to existing detectors (Wang et al., 2023). All models are trained using the training set from the FF++ dataset and subsequently tested on the test set of DiFF (Cheng et al., 2024) dataset (which is unseen during training). DiFF comprises 23,661 pristine facial images and four kinds of high-quality forgery images [Text-to-Image (T2I), Image-to-Image (I2I), Face Swapping (FS), and Face Editing (FE), with a total of 537,466] generated by thirteen state-of-the-art diffusion methods.

Table 2 shows the performance comparisons with three widely used detectors [their results are cited from Cheng et al. (2024)]. It is clear from the table that our proposed DID method achieves remarkable generalization performance on the diffusion-generated facial forgery dataset. DID exceeds the competitor detectors by a large margin on the facial forgery dataset generated with T2I, I2I and FE diffusion techniques. For instance, DID exhibits advantages over DIRE (Wang et al., 2023) which is specifically designed for deepfake detection of diffusion technique-generated images by 81.45% (for T2I), 12.82% (for I2I), and 17.16% (for FE), respectively. Additionally, DID is highly competitive on the forgery dataset generated with FS diffusion technique. These results further demonstrate the superiority of our DID method.

**Table 2:** Cross-dataset evaluation from FF++ to the diffusion-generated DiFF dataset.

**Methods**	**Test subset**			
	**T2I**	**I2I**	**FS**	**FE**
Xception (Rossler et al., 2019)	0.624	0.568	**0.860**	0.586
F^3^-Net (Qian et al., 2020)	0.669	0.676	0.810	0.606
DIRE (Wang et al., 2023)	0.442	0.646	0.850	0.577
DID (Ours)	**0.802**	**0.741**	0.817	**0.676**

The highest results are highlight in bold.

### 4.4 Ablation study

#### 4.4.1 Effect on different training datasets and backbones

To further evaluate the generalization capability of our proposed method under different cross-dataset situations and backbone architectures, we train our DID model on the DFFD dataset (Dang et al., 2020) using two different backbones (ResNet50 and EfficientNet-v2-L) and test its performance on the Celeb-DF test set. DFFD consists of real images and deepfakes generated by various methods, including FaceSwap, Deepfake, Face2Face, FaceAPP, StarGAN (Choi et al., 2018), PGGAN (Karras et al., 2018) (two versions), StyleGAN (Karras et al., 2019), and videos from Deep Face Lab. Experiments are conducted on a subset of DFFD following the protocols established in Dang et al. (2020), excluding inaccessible videos.

As shown in Table 3, DID consistently achieves performance improvement across all backbones fine-tuned with the BCE loss. Notably, it achieves a 17.26% gain in AUC (0.727 vs. 0.620) and a 19.22% improvement in EER (0.763 vs. 0.716) compared to the ResNet-50 backbone. With EfficientNet-V2-L as the backbone, the improvements are 6.56% (0.763 vs. 0.716) on AUC and 12.21% (0.302 vs. 0.344) on EER. These results demonstrate the applicability of our method across different datasets and various feature extraction backbones in the context of cross-dataset deepfake detection.

**Table 3:** Evaluation on DFFD to CDF2 with different backbones.

**Models**	**AUC ↑**	**EER ↓**
ResNet50 + BCE	0.620	0.411
ResNet50 + DID	**0.727**	**0.332**
EfficientNet-v2-L + BCE	0.716	0.344
EfficientNet-v2-L + DID	**0.763**	**0.302**

The highest results are highlighted in bold.

#### 4.4.2 The effect of AUC loss

**Figure 4:**
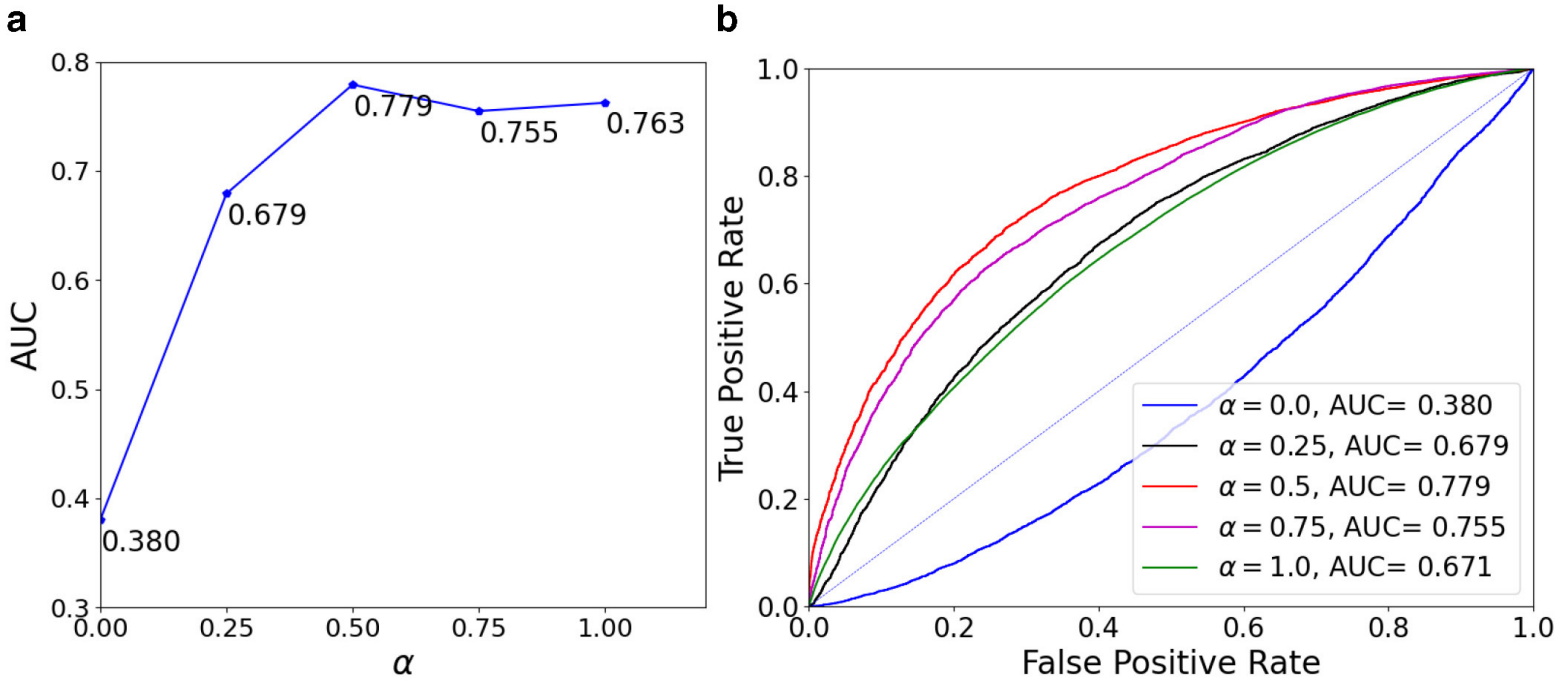
Effect of different α values (used as the balance factor between BCE and AUC loss) on the AUC score. From left to right are: **(a)** is AUC with different α values; **(b)** is ROC with different α values.

The effect of hyperparameter α in the AUC loss, as shown in Equation 9, is analyzed by training our model with varying values of α∈{0.0, 0.25, 0.5, 0.75, 1.0} and presenting the AUC results in Figure 4. When the model is trained using only the AUC loss (α = 0.0) as the deepfake classification loss function, it achieves the lowest AUC score of 0.380. On the other hand, when trained using only the BCE deepfake classification loss (α = 1.0), the model obtains an AUC of 0.763. Interestingly, the model trained with an equal weighting of both the AUC loss and the BCE loss (α = 0.5) delivers the highest AUC score of 0.779, indicating that the AUC loss contributes positively to improving the model's generalization performance.

#### 4.4.3 The effect of *A*_*dom*_ and *T* modules

To investigate the significance of the domain attention module *A*_*dom*_ and the decorrelation learning module *T*, we conducted ablation experiments by training the proposed DID framework with each module removed individually. The detection performance of these models is presented in Table 4. From the results, we observe that when the domain attention module *A*_*dom*_ is excluded from the DID framework (denoted as “w/o *A*_*dom*_” in Table 4), the AUC score drops by 2.05% (from 0.779 to 0.763) and the EER increases by 5.59% (from 0.286 to 0.302) compared to the complete DID model. Moreover, when the decorrelation learning module *T* is removed (denoted as “w/o *T*” in Table 4), the AUC decreases to 0.759, and the EER rises to 0.305, leading to a more significant performance drop than the model without *A*_*dom*_. Specifically, the AUC score decreases by 2.57%, and the EER increases by 6.64%. These results emphasize the necessity of both the *A*_*dom*_ and *T* modules in ensuring the robustness and effectiveness of the DID framework.

**Table 4:** Ablation study of removing (“w/o”) the domain attention *A*_*dom*_ or the decorrelation learning *T* module from the DID framework.

**Models**	**Modules**			**AUC ↑**	**EER ↓**
	*A*_*df*_	*A*_*dom*_	*T*		
w/o *A*_*dom*_	√	×	√	0.763	0.302
w/o *T*	√	√	×	0.759	0.305
DID	√	√	√	0.779	0.286

#### 4.4.4 Analysis of domain classification module

**Figure 5:**
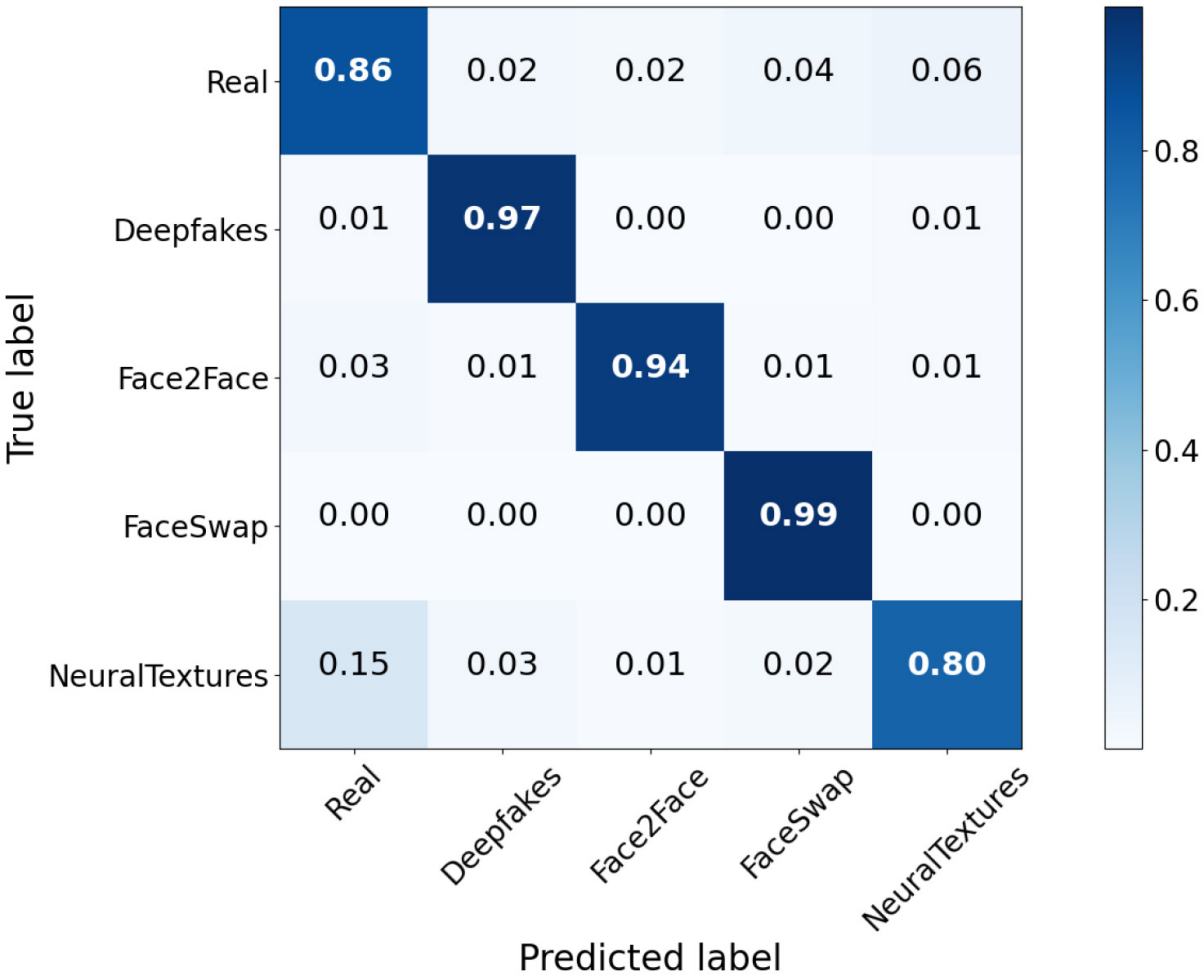
Confusion matrix visualization of domain feature classification. Each deepfake technique is recognized by the domain classification module with high accuracy (the value on the diagonal).

Figure 5 presents the confusion matrix of the domain feature classification. The matrix clearly demonstrates that the domain classification module achieves excellent accuracy in distinguishing various forgery methods. Notably, the average classification accuracy across all methods is 0.91, with the FaceSwap method being classified with the highest accuracy of 0.99. These results indicate that the domain-specific information is effectively separated from the deepfake-relevant features and successfully captured by the domain classification module. This outcome aligns with our decomposition objective and significantly enhances the deepfake detection process.

### 4.5 Visualization

#### 4.5.1 Visualization of the saliency map

**Figure 6:**
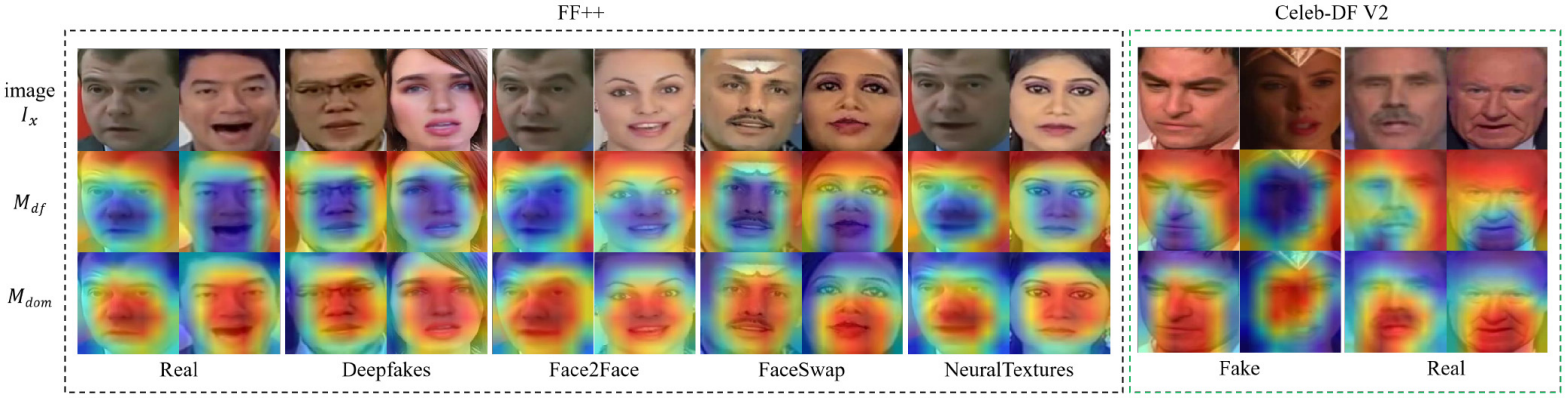
Visualization of the real/fake attention map *M*_*df*_ and the domain (forgery technique) attention map *M*_*dom*_ on the FF++ and CDF2 datasets (images adapted with permission from Rossler, A., 2019, https://www.kaggle.com/datasets/xdxd003/ff-c23, licensed under CC-BY-NC, and from Li Y., 2020, https://www.kaggle.com/datasets/reubensuju/celeb-df-v2, licensed under CC-BY). We can see that *M*_*dom*_ captures the forgery technique-related information (e.g., the forged region) while *M*_*df*_ focuses on the information invariant to forgery techniques.

To provide a more intuitive understanding of our method's effectiveness, we visualize the Grad-CAM outputs of the deepfake attention map *M*_*df*_ and the domain (forgery technique) attention map *M*_*dom*_ in Figure 6. The figure shows that the activation regions of *M*_*df*_ and *M*_*dom*_ differ significantly. *M*_*dom*_ primarily highlights facial regions such as the nose, mouth, and eyes. On the other hand, *M*_*df*_ focuses on the information that remains consistent across different forgery techniques. These visualizations confirm the efficacy of our approach: the decorrelation learning module successfully encourages the disentangled components to contain distinct, independent information, as intended by the design of our method.

#### 4.5.2 Visualization of deepfake features

**Figure 7:**
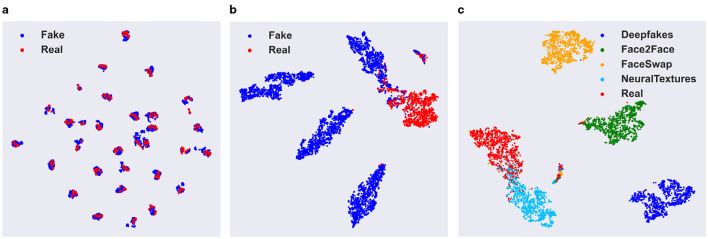
From left to right are visualizations of: **(a)** deepfake features of the EfficientNet-v2-L backbone network; **(b)** our DID framework's deepfake features; **(c)** our DID framework's domain features.

Figures 7a, b present the T-SNE visualizations of the deepfake feature vectors extracted by the backbone network EfficientNet-v2-L and our DID framework, respectively. In both figures, red and blue dots represent real and fake face image features, respectively. The figures show that feature vectors from the backbone network are intermingled in the feature space, indicating poor separation between real and fake features. In contrast, the feature vectors generated by our DID framework are clearly separated in the feature space, demonstrating superior discrimination between real and fake faces. This highlights the effectiveness of DID's deepfake feature representation.

#### 4.5.3 Visualization of domain features

Figure 7c further presents a visualization of the domain features acquired by our DID framework. As depicted in the figure, the domain features extracted from facial images generated by diverse forgery techniques are distinctly separable within the embedding space. The features corresponding to the same forgery approach are closely grouped together, while those from different methods are spaced far apart. These observations validate that our DID framework effectively captures and separates deepfake technique-related information. It should be noted that, although *I*_*dom*_ demonstrates a remarkable ability to discriminate among different forgery techniques as illustrated in Figure 7c, this capability is confined to the forgery techniques that were presented during the training phase. In the case of encountering unfamiliar forged images, *I*_*dom*_ fails to precisely identify the forgery techniques employed in the images and is also incapable of accurately ascertaining the authenticity of the images.

## 5 Conclusion

In this paper, we introduce a deep information decomposition (DID) framework that decomposes facial representations in deepfake images into deepfake-related and unrelated information. The framework further refines these components to ensure clear separation, leveraging only deepfake-related features for distinguishing real from fake images. This approach enhances the deepfake detection model's robustness to irrelevant variations and improves generalization to unseen manipulation techniques. Extensive quantitative evaluations and visual analyses demonstrate the effectiveness and superiority of the proposed DID framework in cross-dataset deepfake detection.

The DID framework is designed with generalizability in mind, making it potentially applicable to other tasks such as pose-, expression-, and age-invariant face recognition, although still possesses certain limitations. Currently, all hyperparameters in the loss function require manual tuning through extensive experimental trials, which is both inefficient and suboptimal. Moreover, the domain classification module depends on access to domain-specific information from the original deepfake datasets, which is often unavailable or incomplete in real-world scenarios.

To address these limitations, several promising directions can be pursued in future work. First, the hyperparameter optimization process could be automated during training to reduce dependence on manual tuning and improve reproducibility. Second, an auxiliary module could be developed to infer or replace the need for explicit domain-specific information, thereby enhancing the framework's adaptability in real-world scenarios where such metadata is scarce or incomplete. These improvements are expected to significantly increase the practicality and robustness of the DID framework for real-time and large-scale deepfake detection.

## Data Availability

The original contributions presented in the study are included in the article/supplementary material, further inquiries can be directed to the corresponding authors.
